# (3,5-Dimethyl-1*H*-pyrazol-1-yl)tri­methyl­silane

**DOI:** 10.1107/S2414314620014443

**Published:** 2020-11-06

**Authors:** Uwe Böhme, Florian Bitto

**Affiliations:** a Institut für Anorganische Chemie, Technische Universität Bergakademie Freiberg, Leipziger Str. 29, 09599 Freiberg, Germany; University of Otago, New Zealand

**Keywords:** crystal structure, *in situ* crystallization, organosilane

## Abstract

The title compound, C_8_H_16_N_2_Si, crystallizes in the the ortho­rhom­bic space group *P*2_1_2_1_2_1_ with one mol­ecule in the asymmetric unit. The Si—N bond is 1.782 (2) Å, which is substanti­ally longer than is found in comparable (3,5-di­methyl­pyrazol­yl)silanes. The tri­methyl­silyl group adopts a staggered conformation with respect to the planar 3,5-di­methyl­pyrazolyl unit. C—H⋯N hydrogen bonds between neighboring mol­ecules form a strand of mol­ecules along the *b-*axis direction.

## Abstract 2

At 1.782 (2) Å, the Si—N bond is substanti­ally longer than is found in comparable (3,5-di­methyl­pyrazol­yl)silanes. The tri­methyl­silyl group adopts a staggered conformation with respect to the planar 3,5-di­methyl­pyrazolyl unit. C—H⋯N hydrogen bonds between neighboring mol­ecules form a strand of mol­ecules along the *b-*axis direction.

## Graphical abstract

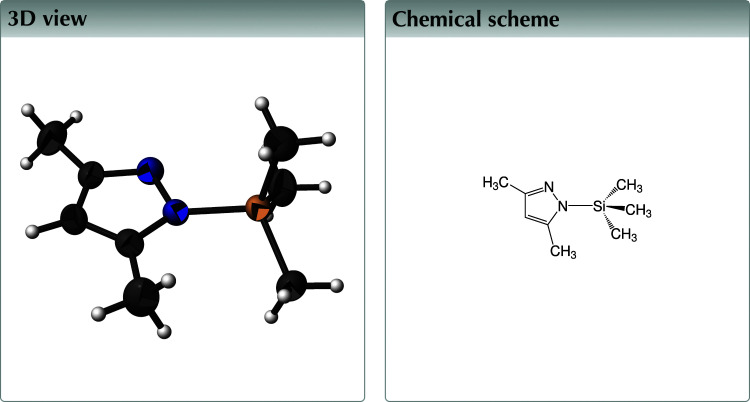

## Structure description

Silanes substituted with 3,5-di­methyl­pyrazolyl units have been investigated over the last twenty years as an alternative to the long-known pyrazolylborates (Pullen *et al.*, 2000 ▸; Kuzu *et al.*, 2008 ▸; Armbruster *et al.* 2009 ▸). Such (3,5-di­methyl­pyrazol­yl)silanes form potent multidentate ligands with a ‘podand topology’ (Gade 2002*a* ▸,*b* ▸,*c* ▸).

The title compound (3,5-dimethyl-1*H*-pyrazol-1-yl)tri­methyl­silane is a key compound in the preparation of different (3,5-di­methyl­pyrazol­yl)silanes by transsilylation (Armbruster *et al.*, 2009 ▸; Bitto *et al.*, 2012 ▸, 2013 ▸, 2016*a* ▸). The solid-state structure of this compound has never been determined, since it is a liquid at room temperature. *In situ* cryo-crystallization of low-melting compounds has been practiced for many years (Atoji *et al.*, 1955 ▸; Smith & Lipscomb, 1965 ▸; Brodalla *et al.*, 1985 ▸). Different *in situ* cryo-crystallization techniques have been described in a review (Boese & Nussbaumer, 1994 ▸). State of the art of *in situ* crystallization is summarized recently in a special issue of *Zeitschrift für Kristallographie* (Boese, 2014 ▸). We have performed several single-crystal structure determinations of pyrophoric liquids by *in situ* crystallization on the diffractometer (Schmidt *et al.*, 2013 ▸; Gerwig *et al.* 2020 ▸). With the experience gained in these processes, we were able to crystallize the title compound on the diffractometer and we report its crystal structure here.

**Figure 1:**
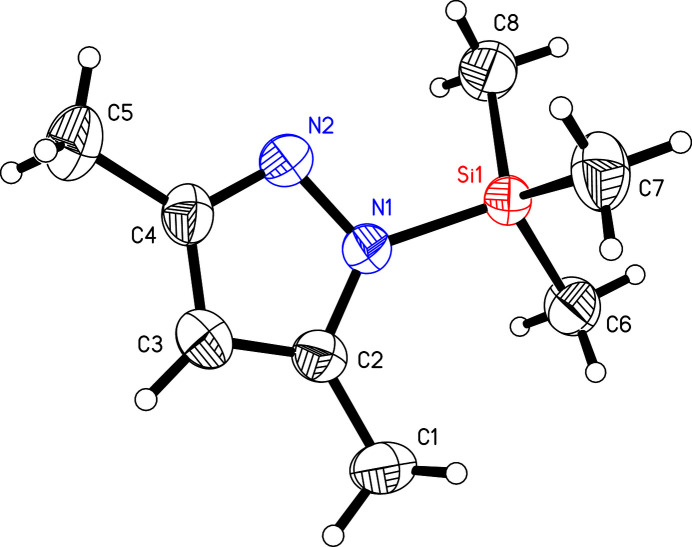
The mol­ecular structure of the title compound, drawn with 50% probability displacement ellipsoids.

**Figure 2:**
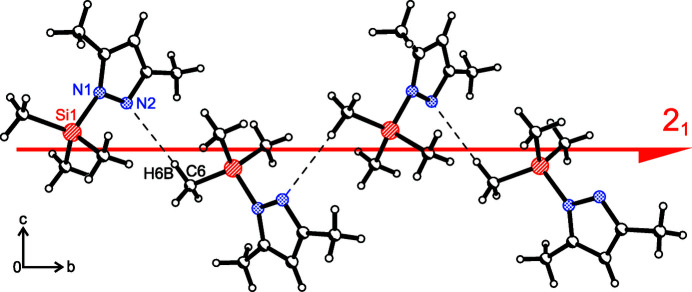
Hydrogen bonds between N2 and H6*B* of neighboring mol­ecules along a twofold screw axis (2_1_) parallel to the crystallographic *b* axis.

The title compound crystallizes in the ortho­rhom­bic space group *P*2_1_2_1_2_1_ with one mol­ecule in the asymmetric unit (Fig. 1 ▸). The Si1—N1 bond is 1.782 (2) Å long. This is substanti­ally longer than comparable bonds in tris­(3,5-di­methyl­pyrazol­yl)methyl­silane [1.745 (5) Å; Vepachedu *et al.*, 1995 ▸] and tetra­kis­(3,5-di­methyl­pyrazol­yl)silane [from 1.712 (3) to 1.725 (3) Å; Armbruster *et al.* 2009 ▸). The pyrazol ring is planar with an r.m.s. deviation of 0.003 Å from the ring plane. The tri­methyl­silyl group adopts a staggered conformation with respect to the plane of the 3,5-di­methyl­pyrazolyl unit. This can be seen in the torsion angles C8—Si1—N1—N2 with −35.1 (2)° and C6—Si1—N1—C2 with 36.3 (2)°. The methyl group C7 is orientated perpendicular to the 3,5-di­methyl­pyrazolyl unit. There is a hydrogen bond between the hydrogen atom at C6 and the nitro­gen atom N2 from a neighboring mol­ecule (see Table 1 ▸). These hydrogen bonds form a strand of mol­ecules generated by a twofold screw axis (2_1_) along the crystallographic *b* axis (see Fig. 2 ▸).

## Synthesis and crystallization

The title compound was prepared from 3,5-di­methyl­pyrazol (19.23 g, 0.2 mol) and chloro­tri­methyl­silane (22.81 g, 0.21 mol). The reaction was performed in 300 ml THF as solvent and in the presence of tri­ethyl­amine (21.25 g, 0.21 mol). Tri­ethyl­amine hydro­chloride is formed during the reaction as a voluminous white precipitate. This precipitate is filtered off. After that the solvent is distilled off *in vacuo*. The title compound is isolated by vacuum distillation at 107°C and 1.3 kPa. It is a colourless liquid (26.97 g, 0.16 mol, 80% yield) (Bitto 2016*b* ▸).

The compound was filled as liquid with 10% *n*-pentane in a glass capillary with 0.5 mm diameter. A single crystal was grown on the diffractometer at 255 K. The data collection was perfomed at a slightly lower temperature in order to have a stable crystal on the diffractometer.

## Refinement

Crystal data, data collection and structure refinement details are summarized in Table 2 ▸.

## Supplementary Material

Crystal structure: contains datablock(s) I. DOI: 10.1107/S2414314620014443/sj4215sup1.cif

Structure factors: contains datablock(s) I. DOI: 10.1107/S2414314620014443/sj4215Isup2.hkl

Supporting information file. DOI: 10.1107/S2414314620014443/sj4215Isup3.cml

CCDC reference: 2041610

Additional supporting information:  crystallographic information; 3D view; checkCIF report

## Figures and Tables

**Table 1:** Hydrogen-bond geometry (Å, °)

*D*—H⋯*A*	*D*—H	H⋯*A*	*D*⋯*A*	*D*—H⋯*A*
C6—H6*B*⋯N2^i^	0.97	2.75	3.670 (3)	159

Symmetry code: (i) 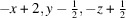 .

**Table 2:** Experimental details

Crystal data	
Chemical formula	C_8_H_16_N_2_Si
*M*_r_	168.32
Crystal system, space group	Orthorhombic, *P*2_1_2_1_2_1_
Temperature (K)	250
*a*, *b*, *c* (Å)	6.1056 (3), 10.8114 (7), 15.5867 (9)
*V* (Å^3^)	1028.88 (10)
*Z*	4
Radiation type	Mo *K*α
μ (mm^−1^)	0.18
Crystal size (mm)	0.50 × 0.40 × 0.40

Data collection	
Diffractometer	STOE *IPDS* 2
Absorption correction	Integration (*X-RED*; Stoe, 2009 ▸)
*T*_min_, *T*_max_	0.708, 0.932
No. of measured, independent and observed [*I* > 2σ(*I*)] reflections	8249, 2346, 2258
*R*_int_	0.048
(sin θ/λ)_max_ (Å^−1^)	0.650

Refinement	
*R*[*F* ^2^ > 2σ(*F* ^2^)], *wR*(*F* ^2^), *S*	0.030, 0.080, 1.09
No. of reflections	2346
No. of parameters	106
H-atom treatment	H-atom parameters constrained
Δρ_max_, Δρ_min_ (e Å^−3^)	0.12, −0.15
Absolute structure	Flack *x* determined using 896 quotients [(*I* ^+^)−(*I* ^−^)]/[(*I* ^+^)+(*I* ^−^)] (Parsons *et al.*, 2013 ▸)
Absolute structure parameter	0.07 (9)

Computer programs: *X-AREA* and *X-RED* (Stoe, 2009 ▸), *SHELXS97* (Sheldrick, 2008 ▸), *SHELXL2014/7* (Sheldrick 2015 ▸) and *ORTEP-3 for Windows* (Farrugia, 2012 ▸).

## full crystallographic data

### Crystal data

**Table:**

C_8_H_16_N_2_Si	*D*_x_ = 1.087 Mg m^−^^3^
*M**_r_* = 168.32	Melting point: 260 K
Orthorhombic, *P*2_1_2_1_2_1_	Mo *K*α radiation, λ = 0.71073 Å
*a* = 6.1056 (3) Å	Cell parameters from 32385 reflections
*b* = 10.8114 (7) Å	θ = 2.3–29.7°
*c* = 15.5867 (9) Å	µ = 0.18 mm^−^^1^
*V* = 1028.88 (10) Å^3^	*T* = 250 K
*Z* = 4	Capillary, colourless
*F*(000) = 368	0.50 × 0.40 × 0.40 mm

### Data collection

**Table:**

STOE IPDS 2 diffractometer	2346 independent reflections
Radiation source: sealed X-ray tube, 12 x 0.4 mm long-fine focus	2258 reflections with *I* > 2σ(*I*)
Plane graphite monochromator	*R*_int_ = 0.048
Detector resolution: 6.67 pixels mm^-1^	θ_max_ = 27.5°, θ_min_ = 2.3°
rotation method scans	*h* = −7→6
Absorption correction: integration (X-RED; Stoe, 2009)	*k* = −14→14
*T*_min_ = 0.708, *T*_max_ = 0.932	*l* = −19→20
8249 measured reflections	

### Refinement

**Table:**

Refinement on *F*^2^	H-atom parameters constrained
Least-squares matrix: full	*w* = 1/[σ^2^(*F*_o_^2^) + (0.0384*P*)^2^ + 0.1226*P*] where *P* = (*F*_o_^2^ + 2*F*_c_^2^)/3
*R*[*F*^2^ > 2σ(*F*^2^)] = 0.030	(Δ/σ)_max_ < 0.001
*wR*(*F*^2^) = 0.080	Δρ_max_ = 0.12 e Å^−^^3^
*S* = 1.09	Δρ_min_ = −0.15 e Å^−^^3^
2346 reflections	Extinction correction: SHELXL-2014/7 (Sheldrick 2015, Fc^*^=kFc[1+0.001xFc^2^λ^3^/sin(2θ)]^-1/4^
106 parameters	Extinction coefficient: 0.038 (8)
0 restraints	Absolute structure: Flack *x* determined using 896 quotients [(*I*^+^)-(*I*^-^)]/[(*I*^+^)+(*I*^-^)] (Parsons *et al.*, 2013)
Primary atom site location: structure-invariant direct methods	Absolute structure parameter: 0.07 (9)
Hydrogen site location: inferred from neighbouring sites	

### Special details

**Table:**

Geometry. All esds (except the esd in the dihedral angle between two l.s. planes) are estimated using the full covariance matrix. The cell esds are taken into account individually in the estimation of esds in distances, angles and torsion angles; correlations between esds in cell parameters are only used when they are defined by crystal symmetry. An approximate (isotropic) treatment of cell esds is used for estimating esds involving l.s. planes.

### Fractional atomic coordinates and isotropic or equivalent isotropic displacement parameters (Å^2^)

**Table:**

	*x*	*y*	*z*	*U*_iso_*/*U*_eq_	
Si1	1.09038 (8)	0.83129 (5)	0.28856 (3)	0.03560 (16)	
N1	0.9829 (3)	0.91025 (15)	0.37963 (9)	0.0371 (3)	
N2	0.8044 (3)	0.98582 (17)	0.36345 (11)	0.0449 (4)	
C1	1.2467 (4)	0.8620 (3)	0.49915 (16)	0.0604 (6)	
H1A	1.2704	0.8905	0.5574	0.091*	
H1B	1.3765	0.8782	0.4651	0.091*	
H1C	1.2171	0.7738	0.4996	0.091*	
C2	1.0558 (3)	0.92883 (18)	0.46120 (11)	0.0390 (4)	
C3	0.9227 (4)	1.01575 (19)	0.49782 (12)	0.0443 (4)	
H3	0.9317	1.0469	0.5540	0.053*	
C4	0.7709 (4)	1.04880 (18)	0.43507 (13)	0.0422 (4)	
C5	0.5894 (5)	1.1411 (2)	0.44085 (18)	0.0670 (7)	
H5A	0.6504	1.2230	0.4493	0.100*	
H5B	0.4951	1.1204	0.4888	0.100*	
H5C	0.5049	1.1397	0.3882	0.100*	
C6	1.2361 (4)	0.6905 (2)	0.32373 (15)	0.0544 (6)	
H6A	1.3721	0.7136	0.3516	0.082*	
H6B	1.2677	0.6390	0.2743	0.082*	
H6C	1.1450	0.6449	0.3637	0.082*	
C7	1.2781 (5)	0.9387 (2)	0.23290 (16)	0.0593 (6)	
H7A	1.1987	1.0127	0.2163	0.089*	
H7B	1.3368	0.8989	0.1821	0.089*	
H7C	1.3972	0.9610	0.2711	0.089*	
C8	0.8569 (4)	0.7920 (3)	0.21896 (16)	0.0589 (6)	
H8A	0.7518	0.7433	0.2511	0.088*	
H8B	0.9084	0.7447	0.1701	0.088*	
H8C	0.7875	0.8675	0.1990	0.088*	

### Atomic displacement parameters (Å^2^)

**Table:**

	*U*^11^	*U*^22^	*U*^33^	*U*^12^	*U*^13^	*U*^23^
Si1	0.0353 (2)	0.0376 (3)	0.0339 (2)	0.0038 (2)	0.0023 (2)	0.00083 (19)
N1	0.0377 (8)	0.0398 (8)	0.0338 (7)	0.0062 (7)	0.0010 (6)	0.0016 (6)
N2	0.0477 (10)	0.0474 (9)	0.0395 (8)	0.0141 (8)	−0.0014 (7)	0.0014 (7)
C1	0.0550 (13)	0.0720 (16)	0.0541 (13)	0.0143 (12)	−0.0176 (10)	−0.0073 (11)
C2	0.0396 (10)	0.0414 (9)	0.0361 (8)	−0.0028 (8)	−0.0016 (8)	0.0000 (7)
C3	0.0515 (11)	0.0435 (10)	0.0380 (8)	−0.0020 (10)	0.0016 (9)	−0.0049 (8)
C4	0.0452 (11)	0.0375 (10)	0.0438 (10)	0.0042 (8)	0.0072 (8)	−0.0007 (7)
C5	0.0701 (16)	0.0590 (14)	0.0718 (16)	0.0264 (14)	0.0064 (14)	−0.0074 (11)
C6	0.0651 (14)	0.0476 (12)	0.0506 (12)	0.0175 (11)	0.0024 (11)	0.0007 (9)
C7	0.0599 (14)	0.0557 (13)	0.0623 (14)	−0.0014 (12)	0.0219 (12)	0.0060 (10)
C8	0.0500 (12)	0.0713 (15)	0.0554 (13)	0.0044 (11)	−0.0075 (10)	−0.0176 (12)

### Geometric parameters (Å, º)

**Table:**

Si1—N1	1.7816 (15)	C4—C5	1.494 (3)
Si1—C8	1.841 (2)	C5—H5A	0.9700
Si1—C6	1.847 (2)	C5—H5B	0.9700
Si1—C7	1.848 (2)	C5—H5C	0.9700
N1—C2	1.362 (2)	C6—H6A	0.9700
N1—N2	1.385 (2)	C6—H6B	0.9700
N2—C4	1.324 (3)	C6—H6C	0.9700
C1—C2	1.494 (3)	C7—H7A	0.9700
C1—H1A	0.9700	C7—H7B	0.9700
C1—H1B	0.9700	C7—H7C	0.9700
C1—H1C	0.9700	C8—H8A	0.9700
C2—C3	1.367 (3)	C8—H8B	0.9700
C3—C4	1.394 (3)	C8—H8C	0.9700
C3—H3	0.9400		
			
N1—Si1—C8	107.15 (10)	C4—C5—H5A	109.5
N1—Si1—C6	109.63 (9)	C4—C5—H5B	109.5
C8—Si1—C6	110.98 (13)	H5A—C5—H5B	109.5
N1—Si1—C7	107.53 (10)	C4—C5—H5C	109.5
C8—Si1—C7	110.39 (13)	H5A—C5—H5C	109.5
C6—Si1—C7	111.02 (13)	H5B—C5—H5C	109.5
C2—N1—N2	109.87 (15)	Si1—C6—H6A	109.5
C2—N1—Si1	133.98 (14)	Si1—C6—H6B	109.5
N2—N1—Si1	115.29 (11)	H6A—C6—H6B	109.5
C4—N2—N1	105.74 (16)	Si1—C6—H6C	109.5
C2—C1—H1A	109.5	H6A—C6—H6C	109.5
C2—C1—H1B	109.5	H6B—C6—H6C	109.5
H1A—C1—H1B	109.5	Si1—C7—H7A	109.5
C2—C1—H1C	109.5	Si1—C7—H7B	109.5
H1A—C1—H1C	109.5	H7A—C7—H7B	109.5
H1B—C1—H1C	109.5	Si1—C7—H7C	109.5
N1—C2—C3	107.29 (18)	H7A—C7—H7C	109.5
N1—C2—C1	123.58 (18)	H7B—C7—H7C	109.5
C3—C2—C1	129.12 (19)	Si1—C8—H8A	109.5
C2—C3—C4	106.16 (17)	Si1—C8—H8B	109.5
C2—C3—H3	126.9	H8A—C8—H8B	109.5
C4—C3—H3	126.9	Si1—C8—H8C	109.5
N2—C4—C3	110.94 (18)	H8A—C8—H8C	109.5
N2—C4—C5	120.6 (2)	H8B—C8—H8C	109.5
C3—C4—C5	128.5 (2)		
			
C8—Si1—N1—C2	156.8 (2)	Si1—N1—C2—C3	169.00 (16)
C6—Si1—N1—C2	36.3 (2)	N2—N1—C2—C1	−179.88 (19)
C7—Si1—N1—C2	−84.5 (2)	Si1—N1—C2—C1	−11.4 (3)
C8—Si1—N1—N2	−35.13 (18)	N1—C2—C3—C4	−0.7 (2)
C6—Si1—N1—N2	−155.65 (15)	C1—C2—C3—C4	179.8 (2)
C7—Si1—N1—N2	83.55 (17)	N1—N2—C4—C3	−0.3 (2)
C2—N1—N2—C4	−0.1 (2)	N1—N2—C4—C5	179.69 (19)
Si1—N1—N2—C4	−171.00 (14)	C2—C3—C4—N2	0.6 (3)
N2—N1—C2—C3	0.5 (2)	C2—C3—C4—C5	−179.4 (2)

### Hydrogen-bond geometry (Å, º)

**Table:**

*D*—H···*A*	*D*—H	H···*A*	*D*···*A*	*D*—H···*A*
C6—H6*B*···N2^i^	0.97	2.75	3.670 (3)	159

Symmetry code: (i) −*x*+2, *y*−1/2, −*z*+1/2.
